# Non-communicable disease risk factors and treatment preference of obese patients in Cape Town

**DOI:** 10.4102/phcfm.v8i1.913

**Published:** 2016-06-10

**Authors:** Kathryn Manning, Marjanne Senekal, Janetta Harbron

**Affiliations:** 1 Division of Human Nutrition, Department of Human Biology, Faculty of Health Sciences, University of Cape Town, South Africa

## Abstract

**Background:**

Insights into the characteristics of treatment seekers for lifestyle changes and treatment preferences are necessary for intervention planning.

**Aim:**

To compile a profile of treatment-seeking obese patients with non-communicable diseases (NCDs) or NCD risk factors and to compare patients who choose group-based (facility-based therapeutic group [FBTG]) versus usual care (individual consultations) treatment.

**Setting:**

A primary healthcare facility in Cape Town, South Africa.

**Methods:**

One hundred and ninety-three patients were recruited in this cross-sectional study. Ninety six chose FBTG while 97 chose usual care. A questionnaire, the hospital database and patients’ folders were used to collect data. Weight, height and waist circumference were measured. STATA 11.0 was used for descriptive statistics and to compare the two groups.

**Results:**

The subjects’ mean age was 50.4 years, 78% were women and of low education levels and income, and 41.5% had type 2 diabetes, 83.4% hypertension and 69.5% high cholesterol. Mean (s.d.) HbA1c was 9.1 (2.0)%, systolic BP 145.6 (21.0) mmHg, diastolic BP 84.5 (12.0) mmHg, cholesterol 5.4 (1.2) mmol/L), body mass indicator (BMI) 39.3 (7.3) kg/m^2^ and waist circumference 117 (12.6) cm). These figures were undesirable although pharmacological treatment for diabetes and hypertension was in place. Only 14% were physically active, while TV viewing was > 2h/day. Mean daily intake of fruit and vegetables (2.2 portions/day) was low while added sugar (5 teaspoons) and sugar-sweetened beverages (1.3 glasses) were high. Usual care patients had a higher smoking prevalence, HbA1c, number of NCD risk factors and refined carbohydrate intake, and a lower fruit and vegetable intake.

**Conclusion:**

Treatment seekers were typically middle-aged, low income women with various modifiable and intermediate risk factors for NCDs. Patients choosing usual care could have more NCD risks.

## Background

Non-communicable diseases (NCDs) such as type 2 diabetes mellitus (DM) and cardiovascular disease (CVD) are among the leading causes of premature morbidity and mortality in developed^1^ countries and developing countries such as South Africa.^2,3^ Within South Africa, it is predicted that the HIV epidemic, which is currently ranked as the leading cause of premature death in the country, may be surpassed by NCDs over the next few decades.^4^ This trend may negatively affect the economic development of the country as the burden from NCDs and obesity is set to immobilise a large proportion of the working-age population.^3,4^

The causes of NCDs are multifactorial; these diseases may arise from any combination of underlying, modifiable, non-modifiable and intermediate risk factors. Research indicates that socio-economic, cultural, political and environmental determinants, including population aging, globalisation, urbanisation and the accompanied nutrition transition contribute to the increase in NCDs in developing countries.^3,5,6,7^ The presence of NCDs and numerous risk factors have been found to be higher in population groups with low income and low educational attainment in both low-middle income and high-income countries.^5,8^ It has also been reported that individuals living in urban environments in South Africa have a high prevalence of modifiable or behavioural risk factors such as an unhealthy diet, physical inactivity, smoking and excessive alcohol intake.^3,6,9^ These modifiable risk factors, together with the presence of non-modifiable risk factors such as age, genetic predisposition, gender and race, result in the development over time of intermediate risk factors such as obesity, high blood pressure, high blood glucose and cholesterol.^10^ The increasing prevalence of obesity worldwide and in South Africa is of particular concern as it exacerbates the other intermediate risk factors and NCDs, resulting in a greater risk for disability and mortality.^11^ The 2003 South African Demographic and Health Survey^12^ reported that 27.4% of women and 8.8% of men aged 15 years and older were obese, which increased to 39.2% of women and 10.6% of men as reported in the 2012 South African National Health and Nutrition Examination Survey (SANHANES).^9^

Both international and national policymakers have called for urgent prioritisation of the prevention and management of NCDs.^3,13,14^ Levitt et al.^4^ suggested that the considerable attention and robust interventions implemented to manage the HIV and AIDS epidemic in South Africa should be applied similarly to NCDs. The restructuring of primary health care (PHC) and district health services in areas of the country such as Cape Town in Western Cape Province to improve standards of care, accommodate the growing population and manage escalating disease burdens^15^ is a step in the right direction. The substantial improvements in NCD surveillance,^3,9^ medication distribution^16^ and policy development^17^ in the country are also encouraging. However, according to Parker et al.,^18^ health promotion, behavioural counseling services and continuity of care in PHC facilities for patients with obesity and NCDs are probably insufficient to address the mentioned lifestyle risk factors. In an attempt to improve this situation, the Western Cape Provincial Department of Health (DoH) developed a NCD management policy that included a six-week, group-based intervention for roll-out at governmental PHC facilities to address modifiable risk factors through behaviour change and treatment adherence.^19^ At the point of roll-out, no research had been done on the socio-demographic, lifestyle and health profiles of patients seeking treatment at these facilities or the potential efficacy. Interestingly, there seems to be a paucity of research on the effectiveness/efficacy of group-based lifestyle interventions in the international literature.^20^ The same is true for research on the characteristics of those who would choose to enroll in such interventions.^20^ Work by Steinsbek et al.^21^ and Galagher et al.^22^ showed that there was little difference between baseline characteristics of participants in group versus standard treatment modalities. However, current literature suggests that patients’ preference for a treatment type for obesity or NCDs in general may be influenced by factors such as age, gender, cultural perceptions, health status, access to quality healthcare and practical difficulties.^23,24,25^ Insights into the characteristics of treatment seekers regarding lifestyle changes and treatment preference are necessary for intervention planning.

The aims of the present research were to compile a socio-demographic, health, lifestyle and stage of change profile of treatment-seeking obese patients with NCDs or risk factors for NCDs attending a district PHC hospital in Cape Town, and to compare these characteristics between patients who chose the group-based programme and patients who chose the usual care option.

## Methods

### Study design

The present paper provides secondary analysis of cross-sectional baseline data of a 6-month lifestyle intervention study where patients could choose to participate in either a 6-week facility-based therapeutic group (FBTG) programme or the usual care option available at a government district PHC hospital in Cape Town, South Africa.

The FBTG intervention comprised six weekly sessions (60 to 90 minutes per week) facilitated by a multi-disciplinary team consisting of a dietitian, medical doctor, pharmacist, physiotherapist and nurse. Education on goal setting, healthy eating, physical activity, behaviour change, adherence to medication and treatment recommendations as well as advice on weight and behaviour maintenance were provided during the six sessions. Six to twelve patients were recruited per FBTG. The usual care group received standard treatment provided to a patient with NCDs and/or risk factors for NCDs at the district PHC hospital. This involves initial one-on-one consultations with the medical doctor and dietitian. Patient-initiated follow-up appointments with the dietitian were possible, pending the availability of the dietitian.

### Study population, setting and sampling strategy

The study population were obese patients attending False Bay Hospital who required management of NCDs or risk factors for NCDs. False Bay Hospital provides medical care for patients without medical health insurance. However, a small minority of patients who have private medical aid are also treated at False Bay Hospital. Patients attending this hospital feed in from suburbs containing formal and informal housing.

Eligible patients were identified by the medical doctors employed by the hospital during routine appointments and were subsequently referred to the hospital dietitian for an individual consultation and possible recruitment. Eligible patients had to be older than 18 years and have a body mass index (BMI) ≥ 30 kg/m^2^, with one or more intermediate risk factor(s) such as raised blood pressure (BP) (> 130/80 mmHg), raised HbA1c (> 7%), raised total cholesterol (> 4.5 mmol/L), or one or more existing NCDs such as type 2 DM or CVD. The doctors made their decision to refer eligible patients to the hospital dietitian based on the most recent biochemical and clinical measures. A basic understanding of the English language was also necessary as both interventions were conducted in English. Patients were excluded if they were pregnant or lactating, or had any form of organ failure, severe psychiatric disorder or physical restrictions. A description of the FBTG intervention was provided during the recruitment consultation with the dietitian, after which eligible patients were given the option to choose the FBTG or the usual care intervention.

The sample size for the cross-sectional arm of the study was based on the sample estimation for the intervention arm. These calculations indicated that 44 to 60 participants would be required in each treatment choice group to achieve 80% power at a 5% significance level. Relevant published results of group-based lifestyle (weight loss) interventions were used for the calculations.^26,27^ As attrition rates in weight loss interventions have been reported to be high (between 10 and 80%),^28^ we recruited a sample including 96 intervention patients and 97 usual care patients, resulting in a total sample of 193 for the development of a profile of patients seeking treatment at the hospital.

### Data collection

Relevant information was obtained using an interviewer-administered questionnaire developed for the purpose of the study, the CLINICOM hospital database and patient folders. During the initial recruitment consultation, the hospital dietitian interviewed the patient to complete the questionnaire and conducted the necessary anthropometric measurements. All biochemical and clinical measures were obtained on the day of recruitment.

#### Socio-demographic information

Socio-demographic characteristics transferred directly from CLINICOM to the data spreadsheet included information on race (coloured, black, white and Asian), gender (male and female), date of birth, family income and residential address of patients. Family income per month was classified using the Western Cape provincial categories, namely H0 for R0.00 and/or government pension, H1 for > R0.00 to R4166.66, H2 for R4166.67 to R8333.33, H3 for > R8333.34, and P for private medical aid. Information collected using the interviewer-administered questionnaire included employment status, number of years of formal education (completed years of schooling, diplomas and certificates) and reason for declining the 6-week FBTG intervention (only asked of patients who chose the usual care option). All patients were also asked to indicate their 6-month weight loss goal.

#### Physical activity assessment

For the physical activity assessment, patients were asked whether or not they participated in formal and/or informal exercise and to specify the exercise type. The frequency of exercise sessions was recorded as the number of times each exercise type was performed per day, per week or per month. The typical duration for each session was recorded in minutes. To calculate the minutes of physical activity performed per week, the number of times the patient exercised was multiplied by seven if daily, divided by one if weekly, and by four if monthly. The subsequent value was multiplied by the indicated duration. If a patient participated in more than one type of exercise, the weekly minutes per week of each exercise were summed. The number of minutes per day spent watching television was recorded and used as a reflection of leisure time sedentary activity.

#### Dietary intake assessment

The focus of the dietary assessment was on determining the number of standard portions consumed per day from indicator food groups. For these purposes, a semi-quantified food frequency questionnaire (FFQ) consisting of 54 food items was developed, from which 10 indicator food groups were derived (Table 1). The indicator food list developed by Seme^29^ for the assessment of the healthfulness of food choices in educators from low socio-economic areas in the Western Cape was adapted for the purposes of the present research by an expert panel of dietitians. The rigorous process followed by Seme^29^ in the development of the food list and the further consideration thereof by the expert panel for application in this study contributed to construct, content and face validity.

**TABLE 1:** Indicator food groups derived from the 54-item FFQ questionnaire.

Indicator food group	Items included (standard portion size)
Fruit and vegetables	Fresh fruit (1 tennis ball size); starchy vegetables (½ cup); cooked vegetables (½ cup), salad (1 cup)
High fat foods	Fried potato chips (1 potato); red meat (90 g portion); organ meat (90 g portion); fried fish (90 g portion); fried chicken (90 g portion); chicken with the skin (90 g portion); pies (1 unit); sausage rolls (1 unit); samoosas (1 portion); vetkoek (1 vetkoek); polony (2 slices); eggs (1); cheese (30 g); full-cream milk (125 ml); take-aways (1 meal)
Fats	Brick margarine (1 tsp); butter (1 tsp); lite margarine (1 tsp); oil (1 tsp); olive or canola oil (1 tsp); mayonnaise (1 tsp); peanut butter (1 tsp)
Energy-dense snacks	Baked goods (1 piece of cake, biscuits); crisps (1 packet); sweets (1 unit); chocolates
High-fibre foods	Brown bread (1 slice); wholewheat or low-GI bread (1 slice); legumes (½ cup); high-fibre cereals (½ cup)
Refined CHO foods	White bread (1 slice); maize porridge (½ cup); sugar (1 tsp); jam (1 tsp); syrup (1 tsp)
Added sugar	White or brown cane sugar added to food or drink (1 tsp)
Sugar-sweetened beverages	Fruit juice (125 ml); sugar-containing carbonated drinks (250 ml); cordials (250 ml)
Lower fat choices	Lean red meat (90 g); poultry without skin or not fried (90 g); tinned fish (90 g or ½ tin); poached or boiled eggs (1); low-fat and fat-free dairy (125 ml); lite margarine (1 tsp); low-fat mayonnaise (1 tsp)
Alcohol	One unit: Beer (340 ml) or tot of spirits (30 ml) or wine (125 ml)

CHO, carbohydrate. *Source*: Adapted from Seme.^25^

The frequency of intake of a standard portion of each food item was recorded as the number of times the food was consumed per day, per week or per month. For quantification purposes, a standard portion size was allocated to each food item and alcoholic or non-alcoholic drinks (Table 1) using the UCT exchange lists and the food-based dietary guidelines for South Africa.^30^ The plate model was used to illustrate standard portion sizes to patients. The number of standard portions consumed per day for each food item was calculated by dividing the number of times the patient consumed the standard portion by one if consumed daily, by seven if consumed weekly, and by 28 if consumed monthly. These values were summed for food items contributing to a particular indicator food group to calculate the daily number of standard portions consumed from each of the 10 indicator food groups. Patients were also asked how many formal meals and snacks they consumed daily.

#### Anthropometric measurements

Anthropometric measurements were conducted according to the World Health Organization (WHO) guidelines.^14^ Weight and height was measured in light clothing and without shoes, using a calibrated scale and stadiometer respectively. BMI was calculated as weight in kilograms (kg) divided by height in metres (m) squared and categorised according to the WHO classification,^31^ namely 30 kg/m^2^ – 34.9 kg/m^2^ as obese class I, 35 kg/m^2^ – 39.9 kg/m^2^ as obese class II, and > 40 kg/m^2^ as obese class III or morbidly obese. Waist circumference was measured midway between the last palpable rib and the top of the hip-bone after normal expiration using a non-stretchable tape measure.^14^ Waist circumference was classified as reflecting increased risk for the development of metabolic syndrome and NCDs if > 102 cm in men and > 88 cm in women.^32^

#### Biochemical and clinical measures, NCD status, medication usage and smoking

Fasting blood samples and BP measurements were collected by nursing staff. The blood samples were analysed by the National Health Laboratory Service (NHLS) for glycated haemoglobin (HbA1c) and total cholesterol. HbA1c was only analysed in patients with type 2 DM. An automated sphygmomanometer was used to measure the BP of each patient when in a seated position with the cuff above the elbow. The diagnosis of hypercholesterolaemia and high BP was confirmed by the medical doctors using the International Classification of Disease codes (ICD-10). Missing values were issued for patients who did not attend their scheduled appointment for biochemical and clinical assessments.

The diagnosis of DM and CVD (specifically ischaemic heart disease, heart failure and peripheral vascular disease) was recorded by the medical doctor in the patient’s folder using the ICD-10 codes. Medication usage was recorded from the medication prescription chart in the patient’s folder. Current smoking status (yes or no and number of cigarettes per day) was determined to calculate the number of ’pack years’ i.e. the number of years an individual had smoked one packet of cigarettes per day.^33^

#### NCD risk factor profile

The risk factor profile calculations described by Van Zyl et al.^6^ were adapted to describe the risk factor profile (total number of risk factors present) in our sample. For these purposes, a score of one was allocated to each of the following: high BP, high cholesterol, current smoking, physical activity < 150 minutes per week,^34^ leisure time sedentary activity > 120 minutes per day,^35^ and fruit and vegetable intake < 5 portions per day.^36^ A minimum of zero indicated no risk factors present while a maximum of six could be obtained if all risk factors were present.

#### Readiness to increase intake of healthy foods

An adapted version of the 12-item Readiness for Change questionnaire (RCQ)^37^ was used to determine patient readiness to increase their intake of healthy foods. The original RCQ was developed to determine the stage of change in individuals with excessive alcohol intake.^37^ This was adapted by Senekal et al. (unpublished) for research on the readiness of members of a South African medical scheme to consume healthy foods. The adapted RCQ aimed to categorise patients in a pre-contemplation (PC), contemplation (C) or action stage (A). The adapted questionnaire consists of the following nine statements, with three relating to each stage of change category:

‘I do not worry about what I eat.’ (PC)

‘I am trying to eat more healthy foods than I used to.’ (A)

‘I enjoy eating unhealthy foods, but eat too much of them at times.’ (C)

‘Eating more healthy foods would be pointless for me.’ (PC)

‘I recently started to eat more healthy foods.’ (A)

‘My intake of unhealthy foods is a problem sometimes.’ (C)

‘There is no need to think about changing what I eat.’ (PC)

‘Sometimes I think that I should eat more healthy foods.’ (C)

‘Anyone can talk about wanting to eat more healthy foods, but I am actually doing something about it.’ (A).

The response categories and scoring were based on the scoring system used by Rollnick et al.,^37^ with a score of -2 allocated to ’Strongly disagree’, -1 to ’Disagree’, 0 to ’Unsure’, +1 to ’Agree’, and +2 to ’Strongly agree’. These scores were summed, with the total score for each stage potentially ranging from -6 to +6. The highest of the three scores reflects the patient’s stage of change. In the event of the scores for two of the three categories being equal, the patient would be classified as being in the higher stage of the two stages.^38^

### Data analysis

Data were entered into a Microsoft Excel (2007) spreadsheet and cleaned prior to analysis in STATA 11.0 (Statacorp Lp, 2009). Numerical variables were checked for normality by exploratory analysis using box and whisker plots and the Shapiro-Wilk test.

Descriptive analyses included calculation of frequencies for categorical data, as well as means (standard deviation) for numerical data. Independent *t*-tests (normal data) and rank-sum tests (non-normal data) were used to compare numerical variables between those who chose the FBTG intervention versus those who chose usual care (referred to as treatment choice groups in the results and discussion). Pearson chi-squared or Fisher’s exact tests (if expected frequencies were < 5) were used to compare categorical variables between the two treatment choice groups. All results with a *p*-value < 0.05 were described as statistically significant.

### Ethical considerations

Permission to conduct the research was obtained from the South African DoH and the medical superintendent of the healthcare facility (False Bay Hospital). Ethical approval was granted by the University of Cape Town’ (UCT) Faculty of Health Science’s Human Research Ethics Committee (Ref: 118/2010). Written informed consent was obtained from each patient.

## Results

### Socio-demographic characteristics

The majority of patients who entered the study were women and of the coloured race (Table 2). The patients were on average 50 years old and had a formal education of 10 years. Most patients indicated that they received a family income between R0.00 and R4166.66, and just over half of the total sample was employed at the time of assessment. There were no significant differences between treatment choice groups for any of these variables (Table 2).

**TABLE 2:** Socio-demographic profile of the total sample and by treatment choice groups.

Profile	Total sample (*n* = 193)	FBTG intervention (*n* = 96)		Usual care (*n* = 97)		*p*-value
	
		*n*	Value	*n*	Value	
**Age in years, mean (s.d.)**	50.4 (12.9)	96	50.3 (13.2)	97	50.4 (12.8)	0.940†
**Gender (column %)**						
Women	77.7	76	79.2	74	76.3	0.631‡
Men	22.3	20	20.8	23	23.7	-
**Race (column %)**						
Coloured	49.2	52	54.2	43	44.3	0.072‡
Black	24.9	17	17.7	31	31.0	-
White or Asian	25.9	27	28.1	23	23.7	-
**Currently employed (%)§**	54.4	48	50.0	57	58.8	0.222‡
**Formal education, mean (s.d.) (years)**	10.1 (2.7)	95	10.5 (2.8)	96	9.7 (2.6)	0.050¶
**Family income per month,†† (column %)**	**(%)**		**(%)**		**(%)**	
H0: (R0.00 or government pension)	19.1	17	17.7	20	20.6	0.671‡
H1: (R0.00 – R4166.66)	61.1	60	62.5	58	59.8	-
H2: (R4166.67 – R8333.33)	12.4	14	14.6	10	10.3	-
H3: (>R8333.34)	4.7	4	4.2	5	5.2	-
Private (medical aid)	2.6	1	1.0	4	4.1	-

s.d., standard deviation.

*n* varies owing to missing values.

† Independent *t*-test (normally distributed data).

‡ Chi-squared test.

§ % ‘Yes’ reported, balance recorded as ‘No’.

¶ Wilcoxon rank-sum test (non-normally distributed data).

†† H0, H1, H2, H3, government classification for monthly income at time of assessment.

The reasons indicated by usual care choice patients for declining entrance into the FBTG intervention included work commitments (*n* = 37; 38.1%), preference for individual consultations (*n* = 14; 14.4%), transport problems (*n* = 9; 9.3%), family commitments (*n* = 5, 5.2%), medical problems (*n* = 2; 2.1%), demotivation (*n* = 1; 1.0%), out of area (*n* = 1; 1.0%) and time limitations (*n*=1; 1.0%). Twenty-seven (27.8%) patients gave no reason for declining the FBTG intervention.

### NCD status, biochemical values, medication usage and smoking

One in 2 patients had one NCD, while approximately 1 in 10 had both DM and CVD (Table 3). Overall, DM was more prevalent than CVD. The majority of patients presented with high BP and high cholesterol. There were no significant differences between the treatment choice groups for these variables (Table 3).

**TABLE 3:** NCDs, biochemical and clinical measures, medication and smoking for the total sample and by treatment choice groups.

Measures	Total sample (*n* = 193)		FBTG intervention (*n* = 96)		Usual care (*n* = 97)		*p*-value
		
	*n*	Value	*n*	Value	*n*	Value	
**NCDs (column %)**							
No NCDs	93	40.4	46	48.0	47	47.4	0.457†
One (diabetes or CHD)	85	51.8	44	45.8	41	42.3	-
Two (diabetes and CHD)	15	7.8	5	5.2	10	10.3	-
**Diabetes (%)††**	81	41.5	36	37.5	45	46.4	0.268†
**CVD (%)††**	35	18.1	18	18.8	17	17.5	0.825†
**NCD risk indicators**							
High cholesterol (%)††	121	69.5	57	65.5	64	73.6	0.249†
HBP (%)††	161	83.4	78	81.3	83	85.6	0.420†
**Biochemical measures, mean (s.d.)**							
HBA1c (%)	65	9.1 (2.0)	31	8.4 (1.5)	34	9.7 (2.3)	0.008‡
Total cholesterol (mmol/L)	166	5.4 (1.2)	83	5.4 (1.2)	83	5.5 (1.6)	0.827§
**Blood pressure, mean (s.d.)**							
Systolic BP (mmHg)	189	145.6 (21.0)	95	142.8 (20.6)	94	148.3 (21.2)	0.127§
Diastolic BP (mmHg)	189	84.5 (12.0)	95	83.6 (11.7)	94	85.5 (12.3)	0.279‡
**Medication**							
Diabetes medication (column %)							
No (diet controlled)	0	0	0	-	0	-	NA
Using oral medication only	55	67.9	21	58.3	34	75.6	0.263¶
Using insulin only	3	3.7	2	5.6	1	2.2	-
Using insulin and oral medication	23	28.4	13	36.1	10	22.2	-
High cholesterol (%)††	58	49.1	23	44.2	35	53.0	0.342†
High BP (%)††	150	93.1	70	89.8	80	96.4	0.087†
**Smoking status (column %)**							
Never smoked	133	58.6	58	60.4	55	56.7	0.028†
Current smoker	31	16.1	9	9.4	22	22.7	-
Previous smoker	49	25.4	29	30.2	20	20.6	-
**Smoking (pack years), mean (s.d.)**	80	23.6 (20.6)	38	20.6 (18.7)	42	26.4 (22.1)	0.213§

NCDs, non-communicable diseases; s.d., standard deviation; BP, blood pressure; HbA1c, glycated haemoglobin.

*n* varies owing to missing values for patients who did not attend their appointment for biochemical or BP measurements, or if a blood test was not conducted.

† Chi-squared test.

‡ Independent *t*-test (normally distributed data)

§ Wilcoxon rank-sum test (non-normally distributed data).

¶ Fisher’s exact test.

†† % ‘Yes’ reported, balance recorded as ‘No’.

The mean HbA1c for all patients with DM was in the poor glycaemic control range,^39^ and the mean total cholesterol, SBP and DBP values were in the high range (Table 3).^40^ Usual care choice patients had significantly poorer glycaemic control than FBTG intervention choice patients. Total cholesterol and BP did not differ significantly between the two treatment choice groups (Table 3).

The majority of patients with DM were using oral hypoglycaemics, with very few of these patients using insulin therapy alone (Table 3). Almost half of the patients with high cholesterol and most of the patients with high BP were taking cholesterol-lowering and anti-hypertensive medication respectively. There were no significant differences in medication usage between the two treatment choice groups. The majority of the sample had never smoked, while about 1 in 6 patients were smoking at the time of assessments. The usual care choice group was significantly more likely to be current smokers. The number of pack years for the total sample was close to 25 years and did not differ between the two treatment choice groups (Table 3).

### Anthropometry

Results for weight status and other anthropometric measurements are presented in Table 4. The mean BMI of the total sample was in the obese class II range.^31^ Both men and women presented with a mean waist circumference above the recommended cut-offs of > 102 cm and > 88 cm respectively.^32^ Furthermore, the waist circumference of the majority of men and women was greater than the recommended cut-offs. The mean 6-month weight loss goal for the total group was approximately 13 kg. There were no significant differences between the two treatment choice groups for any of these variables (Table 4).

**TABLE 4:** Anthropometric measures for total sample and by treatment choice groups.

Measures	Total sample (*n* = 193)		FBTG intervention (*n* = 96)		Usual care (*n* = 97)		*p*-value
		
	*n*	Value	*n*	Value	*n*	Value	
**Weight (kg), mean (s.d.)**	193	102.4 (18.6)	96	101.9 (17.4)	97	103.0 (19.7)	0.863†
**Height (m), mean (s.d.)**	193	1.6 (0.1)	96	1.6 (0.1)	97	1.6 (0.1)	0.292†
**BMI (kg/m^2^), mean (s.d.)**	193	39.3 (7.3)	96	38.9 (6.8)	97	39.8 (7.8)	0.591†
**BMI categories (column %)**							
30–34 kg/m^2^ (obese Class I)	65	33.7	32	33.33	33	34.02	0.562‡
35–39 kg/m^2^ (obese Class II)	56	29.0	31	32.29	25	25.77	-
> 40 kg/m^2^ (obese Class III)	72	37.3	33	34.38	39	40.21	-
**Waist circumference (WC)**							
Women, mean (s.d.)	147	117.3 (12.6)	76	115.3 (12.1)	71	119.4 (12.8)	0.050§
Men, mean (s.d.)	42	115.8 (9.0)	20	117.4 (9.9)	22	114.3 (8.0)	0.384‡
**WC categories**							
Women > 88 cm (%)**¶**	155	98.6	75	98.7	70	98.6	0.961‡
Men > 102 cm (%)**¶**	44	100.0	20	100.0	22	100.0	-
**Weight loss goal**							
Weight (kg), mean (s.d.)	189	12.7 (7.3)	95	13.6 (7.1)	94	11.8 (7.5)	0.076†

kg, kilograms; s.d., standard deviation; BMI, body mass index; WC, waist circumference; cm, centimetres; m, metres.

*n* varies owing to missing values.

† Wilcoxon rank-sum test (non-normal data).

‡ Chi-squared test.

§ Independent *t*-test (normally distributed data).

¶ % ‘Yes’ reported, balance recorded as ‘No’.

### Physical activity

Approximately 1 in 7 patients in the total sample engaged in exercise sessions, with the mean time spent on this being less than half-an-hour per week (Table 5). Time spent watching television amounted to more than 2 hours per day. There were no differences between the two treatment choice groups for these measures (Table 5).

**TABLE 5:** Physical activity and leisure time sedentary activity for total sample and by treatment choice groups.

Physical activity	Total sample (*n* = 193)		FBTG intervention (*n* = 96)		Usual care (*n* = 97)		*p*-value
		
	*n*	Value	*n*	Value	*n*	Value	
**Participates in exercise sessions (%)†**	27	14.1	13	13.8	14	14.4	1.000‡
**Formal physical activity (min/week), mean (s.d.)**	191	20.7 (77.3)	94	26.1 (99.3)	97	15.5 (47.0)	0.993§
**Leisure time sedentary activity (min/day), mean (s.d.)**	189	136.6 (106.0)	92	130.9 (104.8)	97	141.0 (107.3)	0.286§

Min, minutes; s.d., standard deviation.

*n* varies owing to missing values.

† % ‘Yes’ reported, balance recorded as ‘No’.

‡ Chi-squared test.

§ Wilcoxon rank-sum test (non-normally distributed data).

### Dietary intake

The number of standard portions consumed per day from each of the indicator food groups is presented in Table 6. Data for the total sample show that the intake of fats, high fat foods and energy-dense snacks combined was 8.4 standard portions per day. Approximately five teaspoons of added sugar, more than a glass of sugar-sweetened beverage, and approximately 10 standard refined carbohydrate portions were consumed per day. Only three standard high-fibre food portions, 2 standard fruit and vegetable portions (combined) and less than three standard portions of lower fat choices were consumed per day. Alcohol intake was close to half a unit per day in the total sample. Usual care choice patients ate significantly more refined carbohydrates than FBTG intervention choice patients. There were no differences between the two groups for the other dietary intake variables (Table 6).

**TABLE 6:** Daily intake of indicator food groups for total sample and by treatment choice groups.

Food	Total sample (*n* = 193)		FBTG intervention (*n* = 96)		Usual care (*n* = 97)		*p*-value†
		
	*n*	Mean (s.d.)	*n*	Mean (s.d.)	*n*	Mean (s.d.)	
**Indicator food groups**‡							
Fruit and vegetables	187	2.2 (1.5)	93	2.5 (1.8)	94	1.9 (1.2)	0.065
Fruit	187	1.3 (1.3)	93	1.6 (1.5)	94	1.1 (1.0)	0.095
Vegetables	187	0.9 (0.5)	93	0.9 (0.5)	94	0.8 (0.5)	0.127
High fat foods	187	2.3 (1.7)	93	2.1 (1.4)	94	2.5 (1.8)	0.874
Fats	187	4.6 (2.6)	93	4.6 (2.6)	94	4.7 (2.5)	0.454
Energy-dense snacks	187	1.5 (2.0)	93	1.5 (1.8)	94	1.4 (2.2)	0.271
High-fibre foods	187	3.1 (2.8)	93	3.3 (2.8)	94	2.9 (2.9)	0.225
Refined carbohydrate foods	187	10.6 (10.2)	93	9.4 (7.8)	94	12.4 (12.3)	0.030
Added sugar	187	5.1 (8.5)	93	4.0 (5.8)	94	6.2 (10.5)	0.089
Sugar-sweetened beverages	187	1.3 (2.3)	93	1.1 (2.4)	94	1.5 (2.3)	0.070
Alcohol	187	0.4 (3.0)	93	0.6 (4.6)	94	0.1 (0.5)	0.167
Lower fat choices	187	2.4 (2.0)	93	2.6 (2.0)	94	2.3 (2.1)	0.183
**Meals and snacks, mean (s.d.)**							
Number of meals per day	187	2.6 (0.6)	93	2.6 (0.6)	94	2.5 (0.6)	0.712
Number of snacks per day	187	1.2 (1.2)	93	1.4 (1.3)	94	1.0 (1.1)	0.009

FFQ, food frequency questionnaire; s.d., standard deviation.

*n* varies owing to missing values.

† Wilcoxon rank-sum test used for non-normally distributed data.

‡ See Table 1 for description of indicator food groups.

Results indicated that meals were consumed 2.6 times a day while only one snack was consumed between meals on a daily basis (Table 6). Usual care choice patients ate significantly less snacks than FBTG intervention choice patients (Table 6).

### NCD risk factor profile

The mean number of NCD risk factors present in the total group was four out of a maximum of 6 (Table 7). Usual care choice patients had a significantly higher number of risk factors than FBTG intervention choice patients had. The three highest ranked risk factors in the total group and the two treatment choice groups were inadequate physical activity levels, inadequate fruit and vegetable intake, and high blood pressure. Fruit and vegetable intake below the cut-off and, as mentioned, current smoking, were significantly more likely to be present among usual care choice patients (Table 7).

**TABLE 7:** Risk factor profile for total sample and by treatment choice group.

Risk factor (ranked)	Rank	Total sample (*n* = 193)		FBTG intervention (*n* = 96)		Usual care (*n* = 97)		*p*-value
		
		*n*	% yes	*n*	% yes	*n*	% yes	
< 150 minutes/week physical activity	1	183	95.8	89	94.7	94	96.9	0.493†
< 5 fruit and vegetables daily	2	173	92.5	82	88.2	91	96.8	0.025‡
High blood pressure	3	161	83.4	78	81.3	83	85.6	0.420‡
High cholesterol	4	121	69.5	57	65.5	64	73.6	0.249‡
> 2 hours’ TV viewing	5	102	54.0	47	51.1	55	56.7	0.439^‡^
Currently smoking	6	31	16.1	9	9.4	22	22.7	0.012‡

**Total risk factors, mean (s.d.)**		193	4.0 (1.1)	96	3.8 (1.1)	97	4.2 (1.1)	0.008§

Note: These risk factors are in addition to obesity in all patients, as well as a waist circumference in the risk range for the majority of patients.

M, mean; s.d., standard deviation; TV, television.

*n* varies owing to missing values.

† Fisher’s exact test for comparison of FBTG intervention and usual care group patients.

‡ Chi-squared test for comparison of FBTG intervention and usual care patients.

§ Wilcoxon rank-sum test used for non-normally distributed data.

### Readiness to increase intake of healthy foods

The majority of patients (*n* = 130; 68.8%) were in the contemplative stage of change at the time of the study, with 1.6% (*n* = 3) being in the pre-contemplative stage and 29.6% (*n* = 56) in the action stage. There were no significant differences between the two treatment choice groups (Fisher’s exact test *p*-value = 0.326).

## Discussion

Information on the profile of patients seeking treatment for NCDs in PHC settings, as well as characteristics of those who would be willing to enter a group-based intervention such as the Western Cape DoH’s FBTG, is scarce. This may in part be owing to the complexity of psychosocial, biological, ethnic and environmental influences on health-related perceptions, treatment-seeking behaviour and decision making.^23,29,41^ The results of our research provide important insights into this research question.

The majority of our sample was women, despite the fact that men in the study area may also be likely to be at risk for obesity and NCDs. This finding is not uncommon in lifestyle intervention studies and it has been suggested that women seek treatment for obesity and general health problems more often than men do.^25,42,43^ This suggestion is supported by the findings of a recent study in North-West Province in South Africa, namely that men did not actively participate in healthcare-seeking behaviour and wellbeing when compared with women.^25^ Evidence from developed countries shows that men appear to prefer individualised treatment for health problems and a physical activity focus, over group-based weight loss programmes.^42,44^ Our results indicate that the situation may be different in PHC settings in developing countries, with men being equally likely to choose group-based or usual care interventions as women. However, this trend would need to be confirmed by further research in PHC settings.

Although there were no differences in representation of races between treatment groups in our study, black participants were in the minority in both groups, which does not represent the demographics of the patients in the catchment area for the hospital. It has been reported that willingness to participate in weight loss interventions may be influenced by culture-related factors.^45,46^ These studies showed that recruitment rates of black participants into health research and lifestyle programmes were generally low. A number of studies have shown that black South African women may perceive adiposity as a reflection of good health and wealth, while weight loss may be perceived as an indication of illness, especially regarding HIV or AIDS and poverty.^46,47^ Therefore, cultural acceptance of a larger body size may thus contribute to low motivation to lose weight and thus low recruitment rates of black women into weight loss programmes. It can further be speculated that the perception among African-American women that weight loss and lifestyle programmes are not tailored to their personal challenges and cultural preferences^45,48^ may also be true for black women attending the district PHC hospital in our study.

The mean of 10 years of education indicates that our sample (patients attending a PHC facility) on average have not completed secondary level education (12 years of schooling), with no difference between treatment choice groups. The low education level of the total sample may thus affect treatment outcomes and level of engagement in both treatment choice groups. Shankar et al.^49^ state that exposure to education over an individual’s life course is likely to affect the individual’s response to health problems in adult life. Low education attainment has been shown to be associated with reduced treatment-seeking behaviour,^50^ low desire to change, lower levels of health literacy,^48^ and poorer risk perception and external locus of control^51^ which could negatively affect treatment outcomes such as weight loss or adoption of a healthy eating pattern.

The total sample had an average household income of up to R4167.00 per month, with an annual income of up to R50 004.00, which is considerably less than the average annual income of R78 157.00 to R14 3460.00 in Western Cape Province, as reported in the 2011 Census.^52^ Although it has been suggested by the hospital administration that some patients under-report their income in order to qualify for lower hospital rates, the low income was expected as the catchment area for the hospital includes mainly low socio-economic households, education levels were low, and only half of the total sample were employed. It is plausible that low income may reduce treatment-seeking behaviour in general and, if treatment is sought, to opt for a less costly option. The perceived and actual higher cost of healthy eating^53^ may also be a barrier to enrolment in a lifestyle intervention for either treatment group. It is interesting to note that, despite the fact that there was no difference between treatment choice groups for employment status, usual care patients cited work commitments as the main reason for declining the FBTG intervention. It is plausible that unemployed or part-time employed individuals would be more likely to attend a regular FBTG programme or individual dietetic consultations (usual care choice) than employed individuals. However, financial constraints associated with unemployment may preclude them from doing so.

DM was more prevalent in our sample than CVD, with the elevated HbA1c in those with DM, especially patients in the usual care choice group, reflecting poor long-term blood glucose control. High BP and high cholesterol were present in the majority of the sample. Better control of blood glucose and hypertension was expected as pharmacological treatment for the management of these indicators seemed to be in place. However, only half of those with high cholesterol were on statins, possibly because doctors in this PHC hospital applied risk stratification in patient management that involves an initial focus on lifestyle change for patients who have high cholesterol levels, but are not at high risk for CVD. Factors such as poor compliance with treatment, age, weight status, unhealthy diet and physical inactivity might have contributed to the poor control of blood glucose, BP and cholesterol. Furthermore, NCD risk may be increased in the 16% current and 25% past smokers (= 41% ever smokers) in the total group. The Western Cape population is known to have the highest smoking prevalence in South Africa, with 38.5% being ever smokers, which is in line with our results but much lower than the national prevalence of 20.8%.^9^ Patients in the usual care choice group were significantly more likely to be current smokers, and may therefore be at higher risk for NCDs.

Although a BMI in the obese range was expected, it is very concerning that over a third (37.3%) of patients recruited into the study were morbidly obese (BMI > 40 kg/m^2^). Weight loss should clearly be a major priority in the treatment and management of these patients. The estimated mean 6-month weight loss goal of 12.7 kg for the total group (not significantly different between the two treatment choice groups) equates to approximately 0.5 kg per week and is in line with the general recommendation for weight loss of 0.5 kg to 1 kg per week.^54^ If this rate of weight loss is attained, a weight loss of 12.4% can be expected if the initial mean weight of 102.4 kg for the total group is considered. Modest but sustained weight loss of 10% of initial weight may result in substantial improvements in glycaemic control, BP and lipid profiles, if maintained.^55,56^ Weight loss may also contribute to a reduction in waist circumference, which was in the undesirable range for almost all patients in the study sample, that will contribute to further NCD risk reduction and improved management for those with DM and/or CVD as central adiposity is known to exacerbate insulin resistance, dyslipidaemia and hyperglycaemia.^32^ As there were no differences in anthropometric measures between the treatment choice groups, it is possible that weight status did not influence treatment choice in our study sample.

The need for individualised treatment appears to be an important consideration for patients seeking treatment for obesity.^18^ This may explain why the preference for individual consultations was the second reason most cited by patients in the usual care choice group after work commitments for declining participation in the FBTG intervention. This finding is supported by Parker et al.^18^ who found that the majority of patients interviewed in PHC facilities in Western Cape Province opted for individual counselling as their ideal delivery method for therapeutic nutrition education. On the other hand, patients also seem to choose group-based weight loss programmes for professional guidance and peer support.^18,20^ It must, however, be noted that frequent individual counselling for all patients with obesity or other risk factors for NCDs will remain a challenge for health professionals working in PHCs, as both medical^18^ and dietetic^57^ human resources are inadequate to meet the increasing burden of obesity and NCDs.

One of the most concerning findings among the total group of patients in the present study is the combination of the low level of self-reported physical activity and the time spent watching TV (2 hours per day on average), which was equally poor in both treatment choice groups. Time spent watching TV is often used to gauge the level of sedentary activity.^35^ Excessive TV viewing (2–4 hours per day) is considered an unhealthy behaviour as it is known to displace formal moderate physical activity and promote the consumption of unhealthy snacks and beverages, increasing the incidence and mortality from DM and CVD.^35^ The proportion of the sample who were physically inactive was 86%, which is much higher than the national prevalence of inactivity for men (44.7%) and women (47.6%).^58^ It is also higher than the prevalence of 66.5% found in a study of urban dwellers in Free State Province in South Africa.^6^ Furthermore, the reported 21 minutes per week of physical activity in the total sample is much lower than the 150 (for general health) to 300 minutes per week recommended by the WHO for adults aged 18 to 64 years old, to reduce the risk of NCDs.^34^ The causes of low physical activity were not assessed in this sample, although other research has indicated that socio-environmental factors such as poor neighbourhood safety, lack of recreational space, and lack of exposure to physical activity education over an individual’s lifetime are the primary causes.^59^

The present study demonstrated that the dietary intake reflects the ‘Western’ eating patterns that have been associated with an increased risk of developing NCDs.^10,60^ On average, 8.4 standard portions of fats, high fat foods and energy-dense snacks (combined), 1.3 glasses of sugar-sweetened beverages and approximately five teaspoons of added sugar were typically consumed by our sample per day. Furthermore, refined carbohydrates (10.6 standard portions per day) were consumed more regularly than high-fibre foods (3.1 standard portions per day) and the usual care choice group had a higher intake of refined carbohydrates. The patients in the present study reside in urban communities where there is access to a variety of fast-food restaurants and informal vendors who typically sell foods that are energy-dense and high in fat, salt, sugar and refined carbohydrates. Alcohol intake amounted to approximately half a unit per day, which is in line with recommendations of one unit per day for women and two for men.^61^ Although moderate alcohol intake has been associated with decreased CVD risk and DM incidence, excessive alcohol intake may increase the risk for high BP.^62^ It is possible that some patients might have underreported their alcohol intake, which has been found to be a limitation in other South African studies investigating alcohol intake.^63^

Fruit and vegetable intake was low at 2.2 standard portions per day and falls short of the recommendations made by the 2012 South African food-based dietary guidelines that promote a minimum of five portions per day^36^ and also the recommendations of the dietary approaches to stop hypertension (DASH) programme that promotes 8 to 10 portions of fruit and vegetables per 8400 kcal.^64^ Low intakes of fruit and vegetables may increase the burden of disease and reduce survival rates.^36^ Low intakes in South African populations are not uncommon, with primary school educators in lower socio-economic areas in the Western Cape^29^ and South Africans in general^36^ reportedly consuming less than the FBDG recommendation. Intake of low-fat food choices, reflecting healthy lower fat choices over energy-dense, high fat or fried foods, was low at 2.4 standard portions per day. It is known that healthy food choices are affected by their cost, preference and access as well as cultural acceptance, knowledge and perceived control over healthy food choices.^53,65^ It could therefore be argued that health education may not be sufficient to change purchasing and eating behaviours if the cost of healthy foods continues to be unfavourable, while cheap, unhealthy foods are readily accessible in urban communities. In line with findings from educators in low socio-economic areas in Cape Town,^29^ our results show that the patients’ meal patterns were regular, with infrequent snacking. The latter was more frequent in patients who chose the FBTG intervention, but still below two times per day.

Finally, over and above the presence of a high BMI (obesity), the top three risk factors for NCD development in the total group and in each treatment choice group were inadequate physical activity, low fruit and vegetable intake, and high blood pressure. It is important to note that, if the burden attributable (in percentage) to each of these three risk factors plus being obese in terms of death as reported by Norman et al.^66^ are summed, the total attributable burden of death from these four risk factors is 22.5%. Our findings are similar to those found in a cross-sectional study of urban dwellers in Free State Province in South Africa that ranked physical inactivity, high BMI, hypertension, smoking and high cholesterol in descending order as the primary risk factors for NCDs in their study sample.^7^ The mean number of risk factors was close to four in both groups, which was expected as the sample included patients seeking treatment for NCD-related conditions. The significantly higher number of risk factors in the usual care choice group is an interesting observation, especially as it seemed to be as a result of a higher prevalence of smoking and lower proportion of patients achieving five fruit and vegetable portions daily in the usual care group. Jimenez-Garcia et al.^67^ reported that patients who presented with several risk factors such as smoking, high alcohol intake, physical inactivity and poor diet were more likely to be non-adherent to lifestyle guidelines and have fewer contacts with health professionals. It could be argued that usual care patients were less likely to have changed their lifestyle in the past six months and therefore chose the less intensive usual care option. However, this possibility is not supported by the results of our stages of change assessment, as there were no significant differences between the two treatment choice groups for stages of change. It is important to note that the majority of patients in the total sample were in the contemplative stage, indicating that they were not actively changing unhealthy food choices yet may show intention to change behaviour.^68,69^

The findings of the present research need to be considered while bearing in mind the limitations of using self-reported data. The comprehensive process followed in the development of the questionnaire, as well as the fact that all interviews were conducted by the same well-trained dietitian addresses some of these concerns. Although content, construct and face validity of the food list included in the FFQ were ensured, validation of the daily frequency of intake of items in the indicator food groups is recommended. A further limitation is the fact that data for biochemical measures, including HbA1c and cholesterol levels, were not complete, reducing the sample size and therefore power to detect differences between treatment choice groups for these variables.

## Conclusion

The primary aim of the present research was to describe the characteristics of patients seeking treatment for NCDs and/or risk factors for NCDs. The socio-demographic and health profile of this urbanised sample of obese patients with NCDs (DM or CVD) or risk factors for NCDs shows that treatment-seeking patients attending a district PHC hospital in Cape Town were representative of a middle-aged, low income group comprising mostly coloured women. The most prevalent NCD among this sample was DM, followed by CVD, while the majority of patients suffered from hypertension and over two-thirds had high cholesterol despite the availability of pharmacological treatment. The sample was also characterised by low levels of physical activity and poor food choices that may explain the seemingly poor management of the diseases, as well as the intermediate risk indicators, including obesity. Patients choosing the less intensive usual care may have more NCD risks as they had a higher smoking prevalence, HbA1c, number of NCD risk factors and refined carbohydrate intake, and a lower proportion consumed adequate fruit and vegetables.

The baseline patient profile may aid intervention planners, including the DoH, to ensure the planning of appropriate lifestyle interventions. The results clearly point to the importance of including a strong focus on healthy eating and physical activity options in interventions targeted at patients with NCDs or risk factors for NCDs. The inclusion of specific lifestyle advice for the management of hypertension, hypercholesterolaemia and hyperglycaemia is deemed necessary. It is also important that a behaviour change theory or model should be used in planning and implementing such lifestyle interventions. We recommend that future qualitative research should also investigate the reasons for choosing or declining specific weight loss interventions.
